# Comprehensive analysis of gene expression and DNA methylation datasets identify valuable biomarkers for rheumatoid arthritis progression

**DOI:** 10.18632/oncotarget.22918

**Published:** 2017-12-05

**Authors:** Gang Fang, Qing Huai Zhang, Qianqian Tang, Zuling Jiang, Shasha Xing, Jianying Li, Yuzhou Pang

**Affiliations:** ^1^ Laboratory of Zhuang Medicine Prescriptions Basis and Application Research, Guangxi University of Chinese Medicine, Nanning, China; ^2^ Department of Rheumatism, Ruikang Hospital Affiliated to Guangxi University of Chinese Medicine, Nanning, China; ^3^ Department of Zhuang Medicine, The First Affiliated of Guangxi University of Chinese Medicine, Nanning, China

**Keywords:** Biomarker, DNA methylation, GEO, gene expression, therapeutic methods

## Abstract

Rheumatoid arthritis (RA) represents a common systemic autoimmune disease which lays chronic and persistent pain on patients. The purpose of our study is to identify novel RA-related genes and biological processes/pathways. All the datasets of this study, including gene expression and DNA methylation datasets of RA and OA samples, were obtained from the free available database, i.e. Gene Expression Omnibus (GEO). We firstly identified the differentially expressed genes (DEGs) between RA and OA samples through the limma package of R programming software followed by the functional enrichment analysis in the Database for Annotation, Visualization and Integrated Discovery (DAVID) for the exploring of potential involved biological processes/pathways of DEGs. For DNA methylation datasets, we used the IMA package for their normalization and identification of differential methylation genes (DMGs) in RA compared with OA samples. Comprehensive analysis of DEGs and DMGs was also conducted for the identification of valuable RA-related biomarkers. As a result, we obtained 394 DEGs and 363 DMGs in RA samples with the thresholds of |log2fold change|> 1 and *p*-value < 0.05, and |delta beta|> 0.2 and *p*-value < 0.05 respectively. Functional analysis of DEGs obtained immune and inflammation associated biological processes/pathways. Besides, several valuable biomarkers of RA, including BCL11B, CCDC88C, FCRLA and APOL6, were identified through the integrated analysis of gene expression and DNA methylation datasets. Our study should be helpful for the development of novel drugs and therapeutic methods for RA.

## INTRODUCTION

Rheumatoid arthritis (RA) is a common inflammatory disorder induced disease with a morbidity of ∼1% [1]. RA is characterized by the chronic arthritis with the result of irreversible joint damage, and put persistent pain on patients [2]. Besides, RA is a multi factor related disease, which is influenced by age, race, territory, smoking and so on [3]. Marked improvement has been achieved in the classification and treatment of RA, several biologic response-modifying drugs were also proposed for the inhibition of the inflammatory response, however, most patients don’t obtained any remission [4–7]. While, the poor understanding of its mechanisms has limited the development of effective drugs and therapeutic methods.

DNA methylation plays an important role in the development of complex diseases. It has been well known that hper-methylation of tumor suppressor genes’ promoter as a crucial factor for progression of cancers [8–10]. The rapid development of high-throughput sequencing and gene microarray accelerate the research of DNA modification, including DNA methylation, which is closely associated with RA [11–15]. Through genome-wide DNA methylation profiling in RA, Glossop *et al.* [16] identified RA-related methylation changes that distinct in T- and B-lymphocyte populations. Yuan *et al.* [17] also reported important roles of DNA methylation in fibroblast-like synoviocytes for RA progression. Gene expression could be silenced by DNA methylation through adding methyl to cytosine of CG site and prevent binding of transcription factor to specific regions. So combined analysis of gene expression and DNA methylation profiles should be helpful for the understanding of disease mechanisms. There have been many this type studies, particular in cancers, such as Bapat *et al.* conducted integrated analysis of epigenomic and genomic changes by DNA methylation and obtained several novel biomarkers for prostate cancer [18]. Haller also identified SPP1 as an independent prognostic factor for gastrointestinal stromal tumors through combined DNA methylation and gene expression profiling [19]. While, there has none this type of study for RA.

Here, we proposed to obtain novel methylation and expression signatures for RA through the combined analysis of DNA methylation and gene expression datasets from GEO. Intersect analysis obtained several genes that differential expression and methylation in RA samples simultaneously, which should be potential targets. Functional analysis indicated biological processes/pathways participate in the development of RA, including immune and inflammatory related processes.

## RESULTS

**Figure 1:**
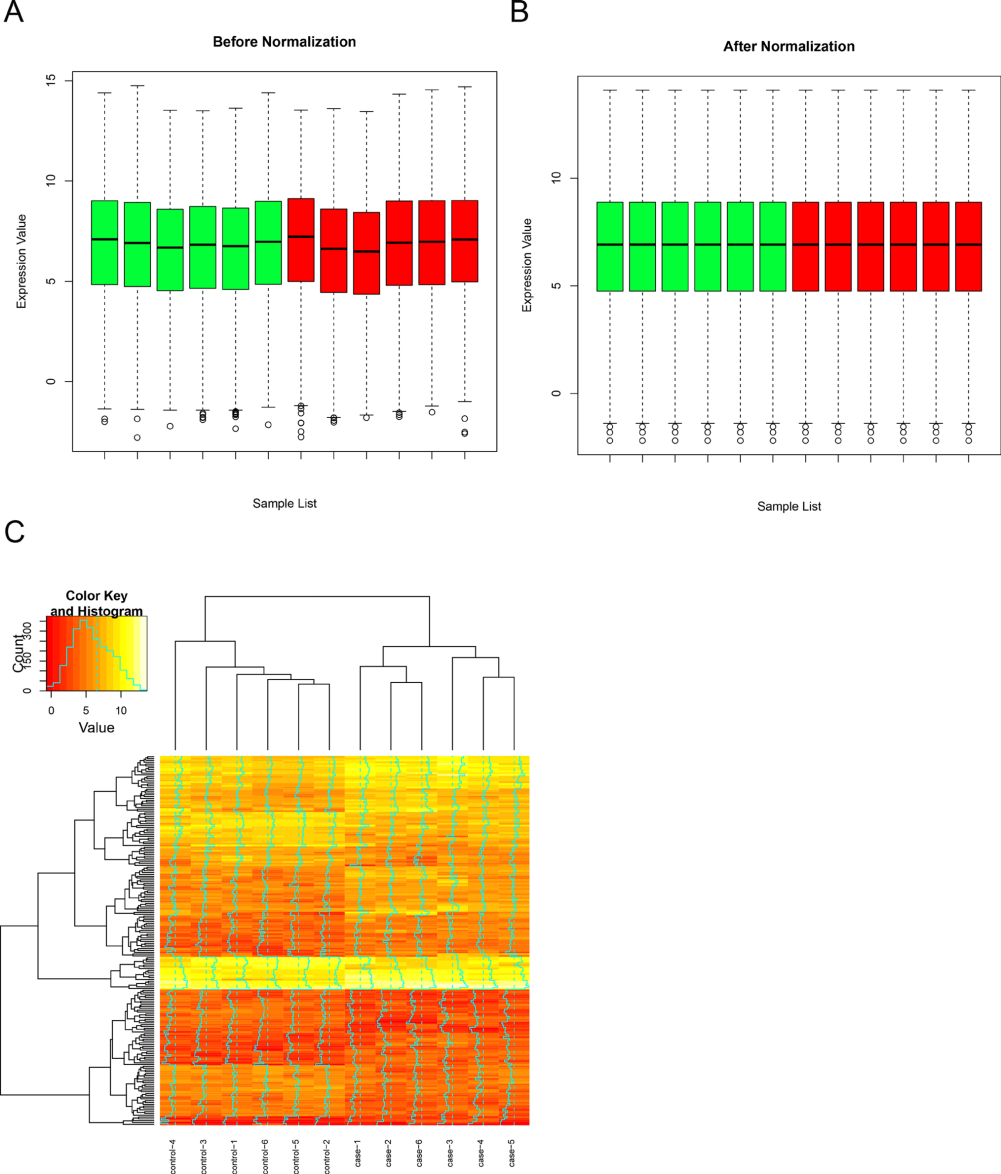
Gene expression microarray analysis Overall expression profiles before (**A**) and after (**B**) normalization. (**C**) Hierarchical clustering of DEGs and samples by euclidean distance. RA and OA samples were clustered into their own group respectively.

DEGs and DMGs Figure 1A and 1B illustrated overall expression profiles in all samples before and after normalization. Significant improvement in the comparable of the expression profiles among all samples was observed, which indicated that the normalized expression values are suitable for the following analysis. We totally obtained 313 DEGs in RA samples compared with OA samples, which including 232 up-regulated genes and 81 down-regulated genes. Hierarchical clustering of DEGs and samples were shown in Figure 1C, in which the control and case samples were clustered into their own groups. Furthermore, 772 differential methylated sites, which correspond to 363 genes, were identified.

### Enriched functions

**Figure 2:**
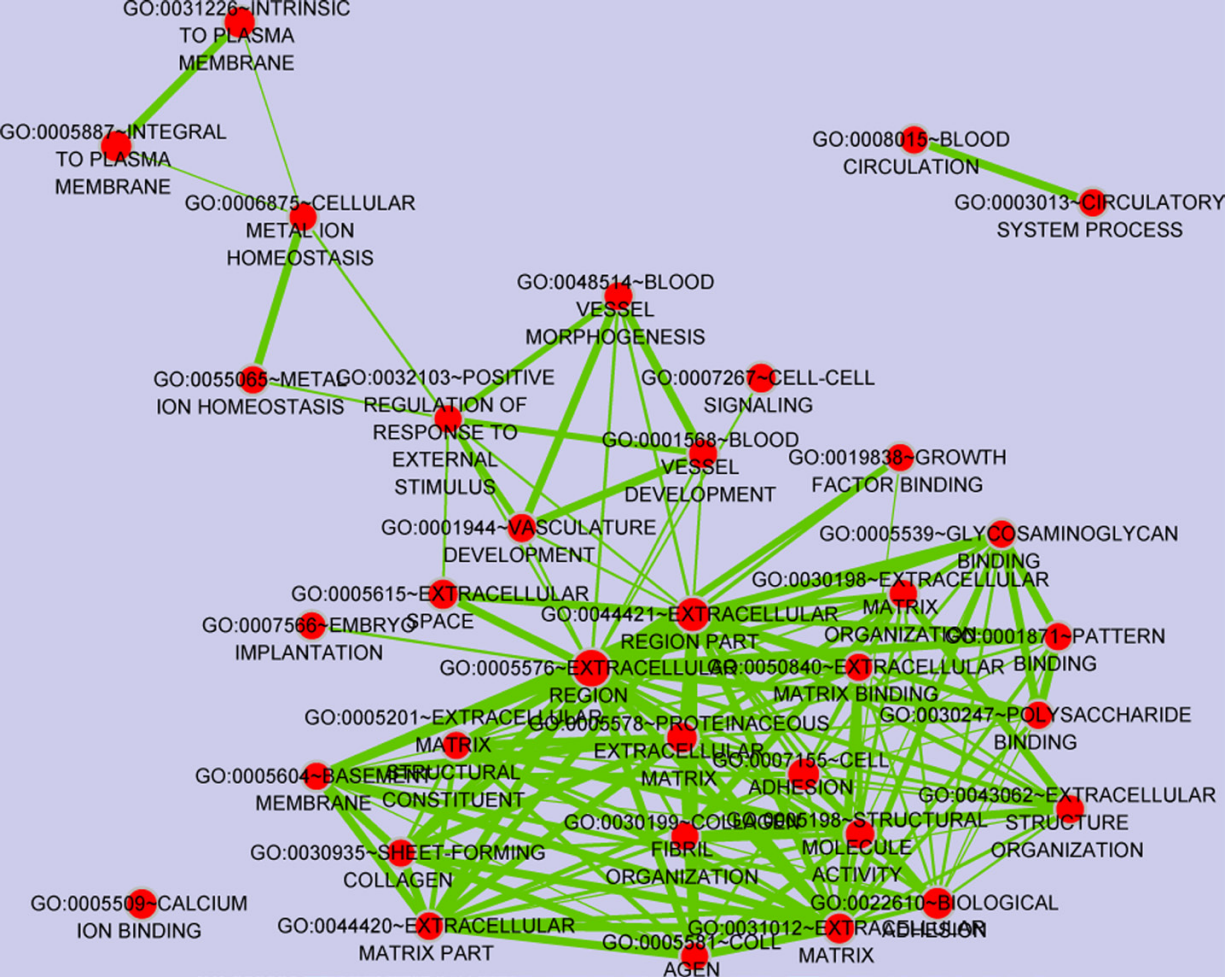
GO terms clustering via the enrichment map plugin of cytoscape software Links between any two GO terms indicates overlapping genes and more thick indicates more overlaps. Node size represents gene number contained in the GO terms and color represents significance.

Functional enrichment analysis identified 60 GO terms satisfied the thresholds of *P*-value < 0.05, which are mainly associated with immune and inflammation. Cluster analysis of GO terms through the enrichment MAP plugin [25] of Cytoscape [26] was shown in Figure 2, in which node size represents gene number contained in the GO term, color represents significance of GO term. Besides, 4 immune and inflammatory related pathways, including primary immunodeficiency, cytokine-cytokine receptor interaction, hematopoietic cell lineage and T cell receptor signaling pathway were also significantly enriched in the DEGs.

### Integrated analysis of DEGs and DMGs

**Figure 3:**
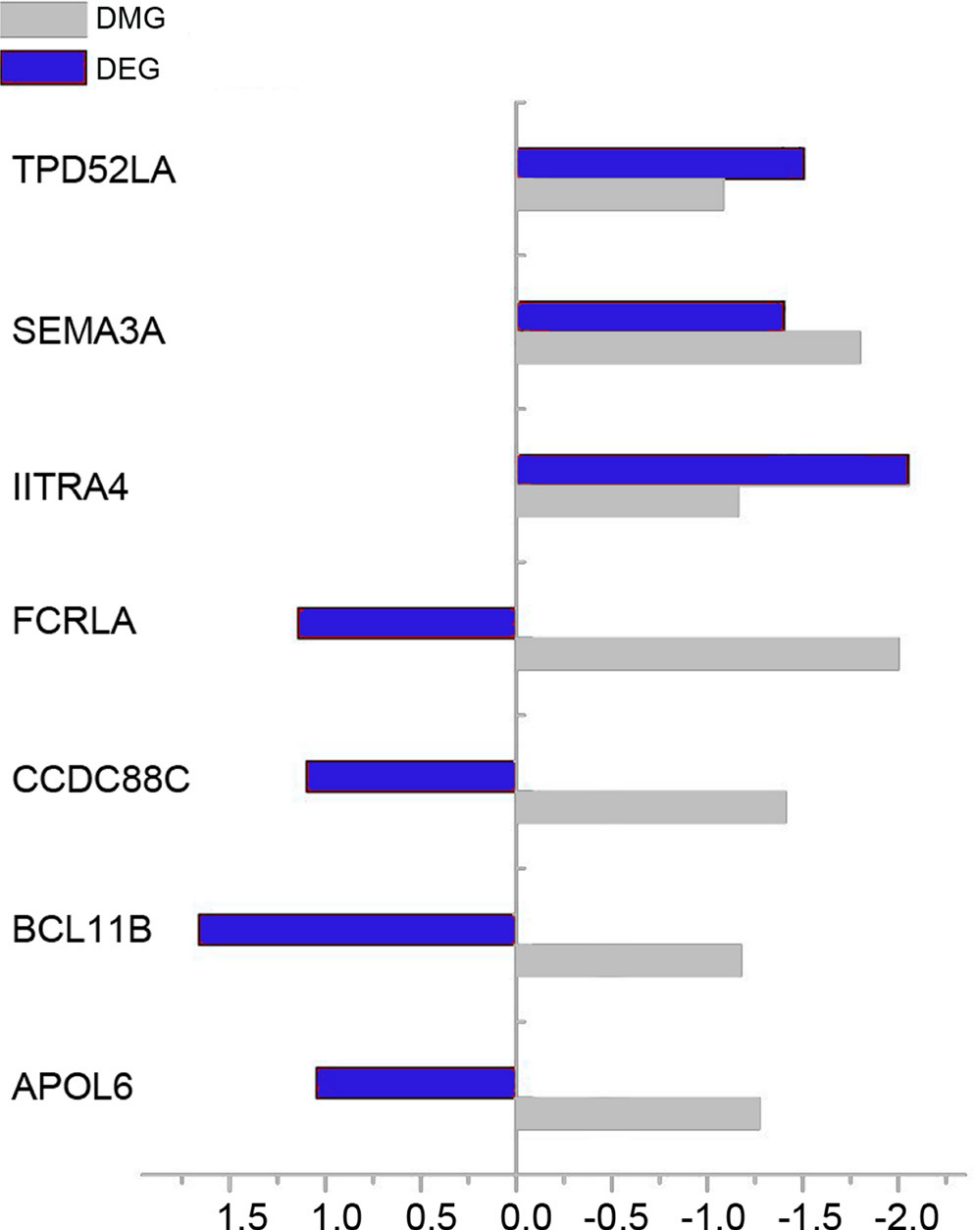
The fold change (log2 scale) of DEGs and DMGs in RA compared with OA samples Blue bar and gray bar represent DEG and DMG respectively.

Intesected analysis of DEGs and DMGs identified 7 overlaps, among which 4 genes were up-regulated and hypo-methylated simultaneously, i.e. APOL6, BCL11B, CCDC88C and FCRLA. Figure 3 illustrated the fold change (log2 scale) of those 7 overlaps.

## DISCUSSION

RA is a multifactorial disease which is also closely associated with genetic. In this study, we performed DNA methylation and gene expression analysis of RA and oA samples to identify potential biomarkers and biological processes/pathways involved in the progression of RA. This should provide comprehensive landscape for RA which is helpful for the development of its diagnosis and treatment.

Functional enrichment analysis of DEGs in RA compared with OA samples identified significantly enriched GO terms and KEGG pathways that are closely related to inflammatory and immune response. It is easy to interpret this results for the close association between inflammatory and immune with RA progression [27–30]. Accordingly, many anti-inflammatory drugs were also developed, such as morin [31], JAK inhibitors [32], TNF inhibitors [33], and so on. Al-Okbi *et al.* even thought that the anti-inflammatory activity of nutraceuticals as a complementary therapy for RA for the severe side effects of anti-inflammatory drugs [34]. Screening of specific targets for drugs has also been an important spot for the treatment of RA.

DNA methylation represents one of the most common epigenetics that could induce gene expression repression through prevent transcription factor from binding the target regions. It is believed that DNA methylation play an important role in RA and several valuable methylation signatures of RA were also obtained [14, 35, 36]. In contrast to most of cancers, in which hyper-methylation is a widespread character, RA tends to be hypo-methylation in its associated tissues and cells [37]. In this study, we obtained 4 genes, i.e. FCRLA, CCDC88C, BCL11B and APOL6, that found to be hypo-methylated and down-regulated in RA compared with OA samples simultaneously. The differential methylation sites in CCDC88C located in promoter region, which should indicate that CCDC88C is more likely participate in the progression of RA. CCDC88C (coiled-coil domain containing 88C) encodes a member of the hook-related proteins that involved in the regulation of the Wnt signaling pathway [38], which could control inflammatory response induced by multiple factors, such as pathogenic bacteria, Toll-like receptor [39, 40]. So we inferred that CCDC88C could influence RA development through Wnt signaling pathway. There is no study about the direct role of APOL6 in RA, while it has been reported that APOL6 could induce immune response in HIV-associated neurocognitive disorders [41]. Besides, APOL6 is also an important apoptosis related protein, which is critical for the progression of RA, so APOL6 should be a novel biomarker in RA.

## MATERIALS AND METHODS

### Microarray datasets

The gene expression profile data was downloaded from GEO with the accession number of GSE36700 that deposited by Toukap [20]. A total of 12 samples were contained, including 5 osteoarthritis (OA) samples and 7 rheumatoid arthritis (RA) samples. The commercial microarray, GPL570 [HG-U133_Plus_2] Affymetrix Human Genome U133 Plus 2.0 Array was used for the quantification of mRNA abundance. The methylation profiles datasets, (accession number: GSE46650), were downloaded from GEO, which was deposited by Rica [21], which including 6 OA samples and 6 RA samples. GPL13534 Illumina Human Methylation 450 BeadChip (HumanMethylation450_15017482) was used for the detection of DNA methylation level and determination of detection *P*-value.

### Data preprocessing

For gene expression datasets, preprocessing of raw microarray, including background correction, quantile normalization and probe summarization of the raw microarray data were all conducted by the affy package [22] of R programming software. For methylation microarray datasets, we performed preprocessing through the IMA package. After beta value normalization and peak correction, the CpG sites with detection *P*-value > 0.05 in more than 75% samples and samples contained more than 75% CpG sites were filtered out. Besides, we mapped the CpG sites to genes through the annotation package.

### Identification of DEGs and DMGs

After preprocessing of raw datasets, DEGs in RA samples compared with OA samples were obtained through the limma package [23] with the thresholds of |log2fold change| > 1 and *P*-value < 0.05. For DNA methylation datasets, we obtained the differentially methylation sites (DMS) firstly through the IMA package with the thresholds of |delta beta| > 0.2 and *P*-value < 0.05. Then, the DMGs were obtained through mapping CpG sites to genes.

### Functional enrichment analysis of DEGs

To explore enriched functions of DEGs, we conducted functional enrichment analysis of DEGs through the Database for Annotation, Visualization and Integrated Discovery, (DAVID) [24]. GO terms and KEGG pathways with FDR < 0.05 was obtained as the enriched functions of DEGs. analysis.

## CONCLUSIONS

In conclusion, this study conducted combined analysis of DNA methylation and gene expression profiles and identified valuable biological processes/pathways and biomarkers for RA. Further studies are still needed for the confirmation of their roles in RA.
